# Shaping the environment – *Drosophila suzukii* larvae construct their own niche

**DOI:** 10.1016/j.isci.2024.111341

**Published:** 2024-11-08

**Authors:** Diego Galagovsky, Ana Depetris-Chauvin, Grit Kunert, Markus Knaden, Bill S. Hansson

**Affiliations:** 1 Max Planck Institute for Chemical Ecology, Department of Evolutionary Neuroethology, Jena, Germany; 2 Max Planck Institute for Chemical Ecology, Department for Biochemistry, Jena, Germany

**Keywords:** Biological sciences, Entomology, Evolutionary biology

## Abstract

In holometabolous insects, the choice of oviposition substrate by the adult needs to be coordinated with the developmental needs of the larva. *Drosophila suzukii* female flies possess an enlarged serrated ovipositor, which has enabled them to conquer the ripening fruit as an oviposition niche. They insert their eggs through the skin of priced small fruits. However, this specialization seems to clash with the nutritional needs for larval development since ripening fruits have a low protein content and are high in sugars. In this work, we studied how *D. suzukii* larvae develop in and interact with the blueberry. We show that despite its hardness and composition, *D. suzukii’s* first instar larvae are able to use the ripening fruit by engaging in niche construction. They display unique physical and behavioral characteristics that allow them to process the hard-ripening fruit and provoke an improvement in its composition that better suits larval nutritional needs.

## Highlights

• D. *suzukii* first instar larvae have massive mouth hooks
• Infestation with larvae improves the nutritional quality of the fruit substrate
• D. *suzukii* larvae prefer transformed, infested fruit tissue
• Their complex digging behavior is consistent with furthering tissue transformation

## Graphical abstract

**Figure:**
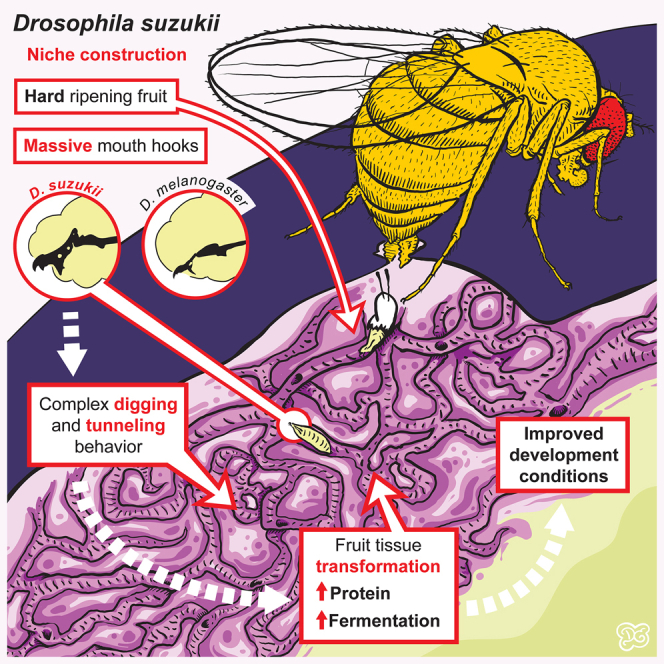

## Introduction

Animals spend significant amounts of energy to generate offspring,^1^ and for natural selection to favor this expenditure it is important to ensure the survival of the upcoming generation. In oviparous animals, new individuals develop helplessly encased in an egg, their fate depending on the oviposition choices of their progenitors. In holometabolous phytophagous and saprophytic insects, this problem is further compounded as development does not end at egg hatching. From the egg, individuals emerge as larvae, which have a limited time to feed and accrue mass in order to enter metamorphosis and become reproductive adults.^2^ Additionally, larval locomotion is energetically costly,^3^ and it is generally expected for the egg-laying individuals to pick oviposition sites that can provide the larvae with the nutrition they will need.^4^

While many *Drosophila* species, including the widely used model organism *Drosophila melanogaster*, have evolved a preference to lay eggs and develop in rotting plant tissue previously settled by yeasts,^5^^,^^6^ *D. suzukii* is also able to use the ripening fruit as an oviposition substrate. They use their enlarged serrated ovipositor to insert the eggs through the skin of small fruits, the likes of blueberry, strawberry, cherry, and grapes.^7^^,^^8^ This physical adaptation has turned them into a growing pest for fruit production.^9^^,^^10^

For *D. suzukii,* occupying this niche has also entailed behavioral adaptations and a preference for the texture, taste, and smell of fresh berries and fruit.^11^^,^^12^^,^^13^^,^^14^^,^^15^ Although they can and will lay eggs on rotting or damaged fallen fruits, they prefer intact fruits.^16^ However, using the intact ripening fruit as an oviposition substrate presents some challenges for the larvae. While for other *Drosophila* species, such as *D. melanogaster*, yeasts and other microorganisms present in fallen rotten fruits provide a source of protein for their development,^5^^,^^17^^,^^18^ the intact ripening fruit is an enclosed environment, with a firm flesh low in protein content and high in sugars.^15^^,^^19^ Moreover, although *D. suzukii* adults prefer to lay eggs on carbohydrate-rich substrates, larvae develop faster on protein-rich substrates, and when given a choice, they prefer more balanced diets, improving their performance.^20^^,^^21^^,^^22^^,^^23^ Hence, the choices of the adult would, in principle, present a disadvantage for the larvae. Nevertheless, *D. suzukii* larvae survive and develop in fresh fruit. How do they do it?

In this work, we sought to describe how first instar *D. suzukii* larvae develop in and interact with a natural substrate, the ripening blueberry. We find that, like the adult flies, *D. suzukii* first instar larvae display physical and behavioral characteristics that allow them to break into the hard ripening fruit, transform and consume it, and further develop in it. We show that as *D. suzukii* larvae tunnel through the substrate, they process it and change its chemical and structural composition, improving it to better suit their nutritional needs. In this way, *D. suzukii* is able to exploit the ripening fruit for its development by constructing its own niche.

## Results

### Developmental progression of *D. suzukii* larvae in blueberries and their physical adaptations

Adult *D. suzukii* female flies are especially adapted to use small ripening fruits as oviposition substrate.^8^^,^^12^^,^^24^^,^^25^^,^^26^ Since they insert their eggs under the skin of the fruit, first instar larvae emerge directly inside the flesh of these fruits, where they must develop. How do larvae survive through development in this hard, sugar-rich, but nutritionally poor medium?

**Figure 1:**
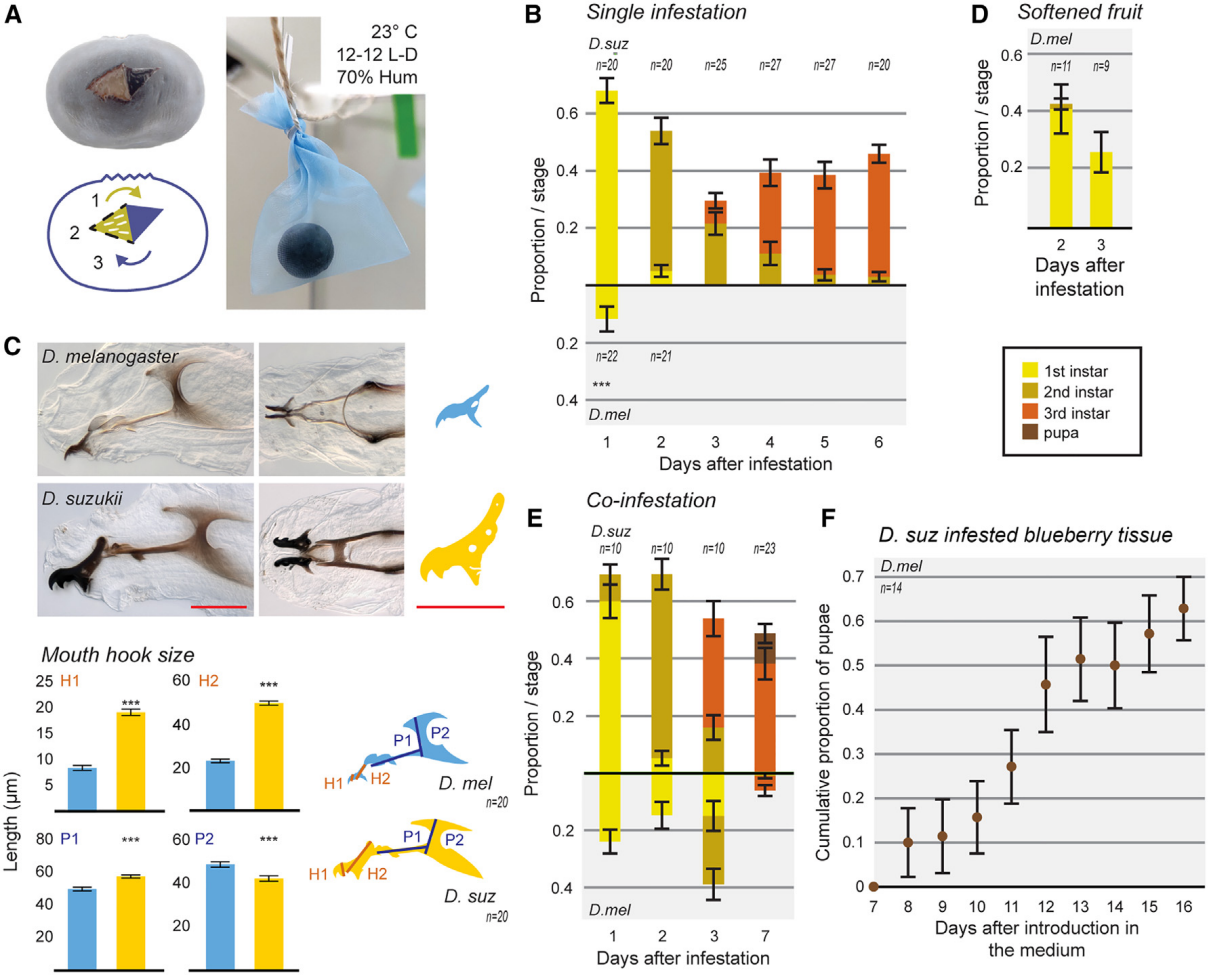
Developmental progression of *D. suzukii* larvae in blueberries and their physical adaptations (A) The procedure developed to study larvae in blueberries. Top, left: Incision into the skin of a blueberry to expose the flesh and place five first instar larvae inside. Bottom, left: Schematics of the procedure. 1. An incision was made to generate and open a flap. 2. Larvae were placed on the exposed fruit tissue. 3. The skin flap was closed. Right: Blueberries in tissue sample bags hanging inside an incubator and the environmental parameters used. (B) Average proportion of five initially placed *D. suzukii* (top) or *D. melanogaster* (bottom), larvae in each developmental stage, found in each fruit at one-day intervals after introduction into fruits (days after infestation). n indicates number of fruits for each treatment. Bars show the means, error bars the SEM. Survival of *Drosophila* larvae of the two species was compared using a binomial generalized linear model (glm), F = 53.059, *p* < 0.001 (∗∗∗). (C) Description of *D. suzukii* and *D. melanogaster* first instar larvae mouth hooks. Top panels: Lateral (left column) and bottom view (mid column) of the mouth hooks of *D. melanogaster* (top row) and *D. suzukii* (bottom row). On the right, enlarged isolated tracings of the structures are shown for a clearer comparison (Scale bar: 20 μm). Bottom graphs: Bar graphs show the mean length of the first tooth from the top of the mouth hook (H1), the length from the top of the mouth hook hinge to the bump posterior to the first tooth (H2), length of the H piece (P1), and vertical length from the top of the dorsal posterior process to the joining of the bottom posterior and anterior processes, passing through the end of the H piece (P2). Bars indicate the mean and error bars the SEM. n indicates the number of mouth hooks analyzed. The size of mouth hook parameters between the two *Drosophila* species was compared with ANOVAs, H1: F = 427.80, H2: F = 1938.00, P1: F = 165.00, P2: F = 32.09, p for all comparison <0.001 (∗∗∗). (D and E) Average proportion of the initially placed larvae of *D. suzukii* or *D. melanogaster* in each developmental stage, found in each fruit at one-day intervals after introduction into fruits (days after infestation), (D) *D. melanogaster* in softened blueberries and (E) Co-infestation experiments. Fruits and larvae were scored independently and discarded at each time point. Bars represent means, error bars SEMs. n indicates the number of larvae containing fruits. (F) Average (mean) cumulative proportion of the initially placed larvae of *D. melanogaster* that pupate at one-day intervals after introduction into vials with infested blueberry tissue from 4 days *D. suzukii* infested blueberries. The same vials were scored each day for the presence of pupae. Error bars: SEM. n represents the number of vials scored for pupae.

As a first step to approach this question, we studied the progression *of D. suzukii* larval development within fresh blueberries, a hard-skinned fruit that adult female flies would typically choose to lay eggs in. We contrasted it to *D. melanogaster* development under the same conditions since this species presents very different egg-laying preferences.^15^^,^^27^ To bypass the need for adults to oviposit in the fruits, we manually infested isolated blueberries with first instar larvae. Thereafter, we kept the berries in porous bags hanging from strings (Figure 1A), which simulates the way they hang on a plant. This allows air to circulate around the fruit and impedes the accumulation of moisture and molding.

In this semi-natural condition, *D. suzukii* first instar larvae were able to progress through development, transitioning to the second instar two days after infestation and reaching the third instar by the fourth day (Figure 1B). In the case of *D. melanogaster*, all larvae died after one day (Figure 1B).

While staging the larvae during development, we noticed that the mouth hooks of first instar *D. suzukii* larvae looked darker and bigger than those of *D. melanogaster*. We found that they differ significantly in the size of several morphological mouth part parameters with the actual hook being significantly bigger in *D. suzukii* than in *D. melanogaster* (Figure 1C), even though both species are similar in mass at this stage (Figure S1B)*.* Thus, like *D. suzukii* adult female flies that have an enlarged ovipositor enabling them to pierce the tough skin of these fruits, *D. suzukii* first instar larvae have massive mouth hooks that may facilitate digging in hard substrates. Interestingly, we observed this same type of massive mouth hooks in the closely related species *Drosophila subpulchrella* (Figure S1A), whose female adults behave similarly to *D. suzukii* and lay eggs through the skin of ripening fruits using their enlarged serrated ovipositor and whose larvae can also develop into adults in ripening fruits.^8^ On the other hand, the mouth hooks of first instar larvae of *D. biarmipes*, an evolutionarily closely related species, whose adult females lack an enlarged ovipositor and therefore cannot lay eggs through the skin of fruits, are similar to the mouth hooks of flies that develop on soft substrates, namely *D. melanogaster* and the tree sap-eating larva of *D. virilis* (Figure S1A).

To test if mechanical barriers could underly the failure of *D. melanogaster* first instar larvae to survive in fresh hard fruit, we softened the fruit tissue manually, which resembles the hardness of an optimized fly rearing medium (Figure S1C) and observed whether this would aid them in developing further in the fruits. In effect, *D. melanogaster* first instar larvae could live longer in these softened fruits, although development did not progress past the first or second instar (Figure 1D). This is an indication that substrate hardness may be an important aspect of the fruit that larvae need to overcome to survive in blueberries but that there are also other crucial factors at play.

We next wondered whether something in the specific interaction of *D. suzukii* larvae with the fruit could be what facilitates larval survival. To test this idea, we co-infested fruits with both species simultaneously. Indeed, under these conditions, *D. melanogaster* larvae were able to reach the third instar by the seventh day post-infestation (Figure 1E). The presence of *D. suzukii* in the blueberries improves the chances of survival of *D. melanogaster* larvae, although still far from those in optimum rearing conditions (Figure S1D).

To further investigate this idea, we asked whether tissue coming from *D. suzukii* infested blueberries would be enough to sustain *D. melanogaster* development. To this end, we isolated the soft, dark fruit tissue that surrounded the larvae from 4-day post-*D. suzukii* infestation blueberries (we call this *infested* *tissue*). We placed five first instar *D. melanogaster* larvae on these tissue samples and left them to develop. In this condition, an average of 62.9% of *D. melanogaster* larvae pupated (SEM = 0.07, *n* = 14) (Figure 1F) and of those an average of 76.0% developed into adult flies (SEM = 0.09, *n* = 14).

Taken together, these results show that the activity of the *D. suzukii* larvae on the fruit renders the fruit more conducive to larval development not only for its own species but also for *D. melanogaster*. Interestingly, our observation that *D. suzukii* larvae possess massive mouth hooks opens the exciting possibility of a mechanical softening of the substrate.

### ***D. suzukii*****larval activity elevates the protein content of the fruit, increasing its physiologically perceived nutritional value**

**Figure 2:**
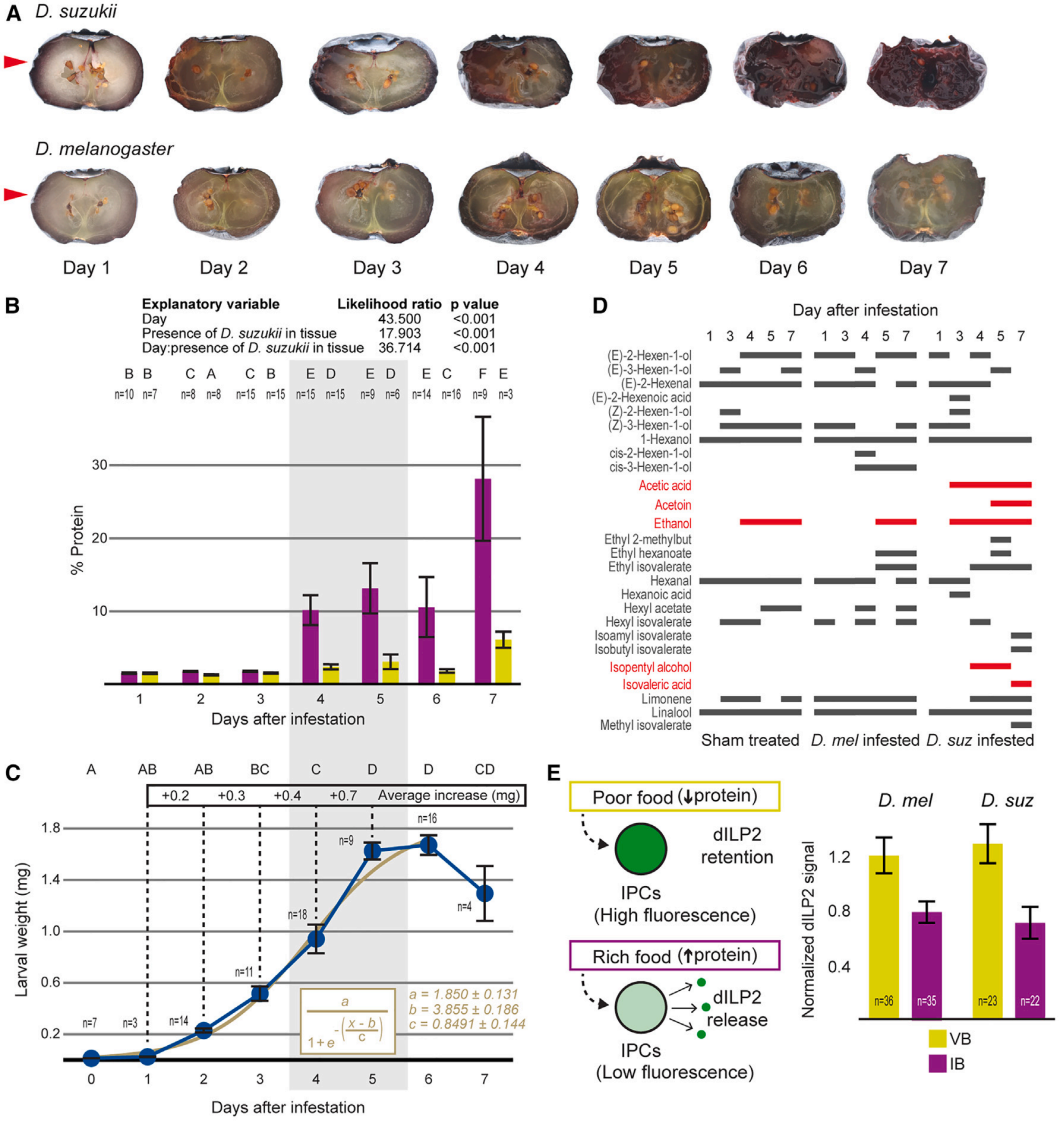
*D. suzukii* larval activity modifies the composition of the fruit, elevates its protein content, and increases its physiologically perceived nutritional value (A) Qualitative changes in blueberries at 1-day intervals after the introduction of *D. suzukii* larvae (top row) or *D. melanogaster* larvae (bottom row). The red arrowhead marks the introduction point. (B) Percentage of protein in sample tissue from single fruits either displaying no signs of larval activity (virgin fruit tissue – green bars) or showing signs of larval activity (infested tissue – purple bars) at one-day intervals after placement of first instar larvae in fruits (days after infestation). The protein content of fruits was analyzed using generalized least squares models. Common uppercase letters on top of the graph indicate non-significant differences between treatments. Bars represent the mean, and error bars the SEM, and n the number of investigated fruits. (C) Progression of the average larval weight (mg) at 1-day intervals during development in blueberries from recently hatched *D. suzukii* first instar larvae. Blue circles represent the mean, error bars the SEM, n the number of investigated samples of larvae. The beige curve represents the 3-parameter sigmoidal growth curve with the equation shown in the graph. All three parameters of the equation are statistically significant (a: t = 14.080, b: t = 26.049, c: t = 5.912, p for all parameters <0.001). (D) Volatile compounds emitted by individual blueberry samples on different days after sham-treated (left), *D. melanogaster*-infested (center), and *D. suzukii*-infested (right) blueberries. Bars: Presence of a chemical in a sample. Red bars: Chemicals of interest exclusively present in *D. suzukii*-infested samples. (E) Schematics of expected dILP2 immunofluorescence signal dynamics in IPCs of larvae feeding on poor or rich protein media (left). Normalized immunofluorescence signals of anti-dILP2 in IPCs of larvae feeding on virgin blueberries (VB - green bar) or 4-day *D. suzukii*-infested blueberries (IB - purple bar) (right) Bars represent the means, error bars the SEM, and n the number of larvae investigated. two-way ANOVA with tissue type and *Drosophila* species as explanatory variables (Tissue type: F = 17.405, *p* < 0.001 (∗∗∗), *Drosophila* species: F = 0.077, *p* = 0.782, Tissue type: *Drosophila* species: F = 1.074, *p* = 0.302).

As *D. suzukii* infestation of the fruit progresses, the visual and textural aspects of fruit tissue change, expanding from the point of infestation. The fruit turns softer to the touch and, from the third day, also darker (Figures 2A and S1E). Additionally, from day 4, bubbles appear on the tissues, and the smell of the fruits turns intensely acidic. Conversely, *D. melanogaster* infestation does not lead to any of these changes in the fruit (Figures 2A and S1E).

Fresh ripening blueberries have a relatively low protein content and are high in sugars.^19^ Such a dietary combination is detrimental to *D. melanogaster* development,^28^^,^^29^ where larvae usually feed on overripe, rotting fallen fruit,^18^ with a higher protein content.^21^ Since *D. melanogaster* larvae develop better on blueberry tissue infested by *D. suzukii*, we investigated the protein content in these fruits, particularly how it is associated with *D. suzukii* presence.

As *D. suzukii* infestation progresses, the blueberry turns into a patchy environment with two distinctive types of tissue; infested blueberry tissue showing signs of larval activity and processing (softening, darkening, presence of larvae) and virgin blueberry tissue yet untouched by larvae (Figure 2A). While in virgin blueberry tissue, unprocessed and untouched by larvae, protein content stays quite low throughout larval occupancy of the fruit, in infested blueberry tissue, the protein content increases dramatically between day 3 and 4 post-infestation, coinciding with the time when the tissue starts displaying the most striking qualitative changes (Figure 2B).

Moreover, it is at this time point, between Day 3 and 4, that larvae have transitioned from the second to the third instar (Figure 1B). In *Drosophila*, the third larval instar is a crucial point for larval growth when most of the mass gain occurs.^30^^,^^31^ Concurrently, *D. suzukii* larvae mass accrual rate is at its highest when the fruit protein content increases (Figure 2C). Our observations suggest that the activity of *D*. *suzukii* larvae changes the fruit composition, coinciding with the time when larval mass gain is at its highest. Interestingly, the protein content and the extent of tissue modification continue to change even past the critical period of larval development, when larvae start switching their behavioral program and stop feeding (Day 7).

In our qualitative assessment of the changes that the fruit undergoes during the infestation, we noted a strong acidic odor emanating from the fruit. This prompted us to analyze the headspace profile of the fruits at different time points in the infestation process. To determine which volatile chemicals are associated with *D. suzukii* infestation, we compared these fruits to *D. melanogaster-*infested and sham-treated uninfested blueberries.

In *D. suzukii*-infested fruits, acetic acid, isopentyl alcohol, and acetoin were distinctly detected between day 3 and 5 post-infestation, when the fruit started displaying noticeable qualitative changes and an increase in protein content (Figure 2D). On the contrary, these three chemicals were not observed in sham-treated and *D. melanogaster*-infested fruits. Interestingly, these volatile chemicals are indicators of microbial activity and fermentation,^32^^,^^33^ pointing toward the involvement of microbes in the transformation of the fruit and its increase in protein content.

The activity of *D. suzukii* larvae on fruit tissue leads to an increase in its protein content and the production of fermentation products. This turns an otherwise nutritionally poor food source into a richer growth medium that can support the mass increase and development of *D. suzukii* as well as of *D. melanogaster*. The question is whether the larvae perceive this change in substrate quality.

From *D. melanogaster,* it is known that in the larval brain, insulin-producing cells (IPCs) produce and secrete several *Drosophila* insulin-like peptides (dILPs), which regulate metabolism and promote growth. The secretion of some dILPs is regulated by the quality of the nutritional input, most specifically, by the levels of dietary amino acids in it. Starved larvae or larvae fed in a protein-poor medium will retain dILPs in the IPCs, while larvae fed on a protein-rich diet will release them^34^^,^^35^^,^^36^ (Figure 2E). Therefore, immunoreactivity levels of dILPs in the IPCs could be used as a proxy to determine the perceived nutritional quality of the feeding substrate.^34^ We thus analyzed how third instar *D. suzukii* or *D. melanogaster* larvae perceive the nutritional value of *D. suzukii*-infested fruit by analyzing dILP release or retention from their IPCs. Both *D. melanogaster* and *D. suzukii* third-instar larvae that fed on *D. suzukii*-infested fruit had lower levels of dILP2 inside the IPCs than larvae fed on uninfested virgin blueberries (Figures 2E and S1F). Therefore, when larvae feed on *D. suzukii*-infested fruits, their brain IPCs release more dILP2. Our results suggest that during the period of maximum growth rate (the third instar), larval physiology in both species perceives the *D. suzukii*-infested fruit tissue as a nutritionally richer food source than the virgin fruit tissue. This is consistent with the observed increase in protein content in this feeding substrate (Figure 2B).

### *D. suzukii* larval behavior is conducive to niche construction

Larval activity modifies the fruit substrate, increasing its protein content concomitantly with the progression of larval development. We therefore wanted to see how larval behavior might lead to the modification of the substrate and test whether larvae respond behaviorally to take advantage of these modifications.

**Figure 3:**
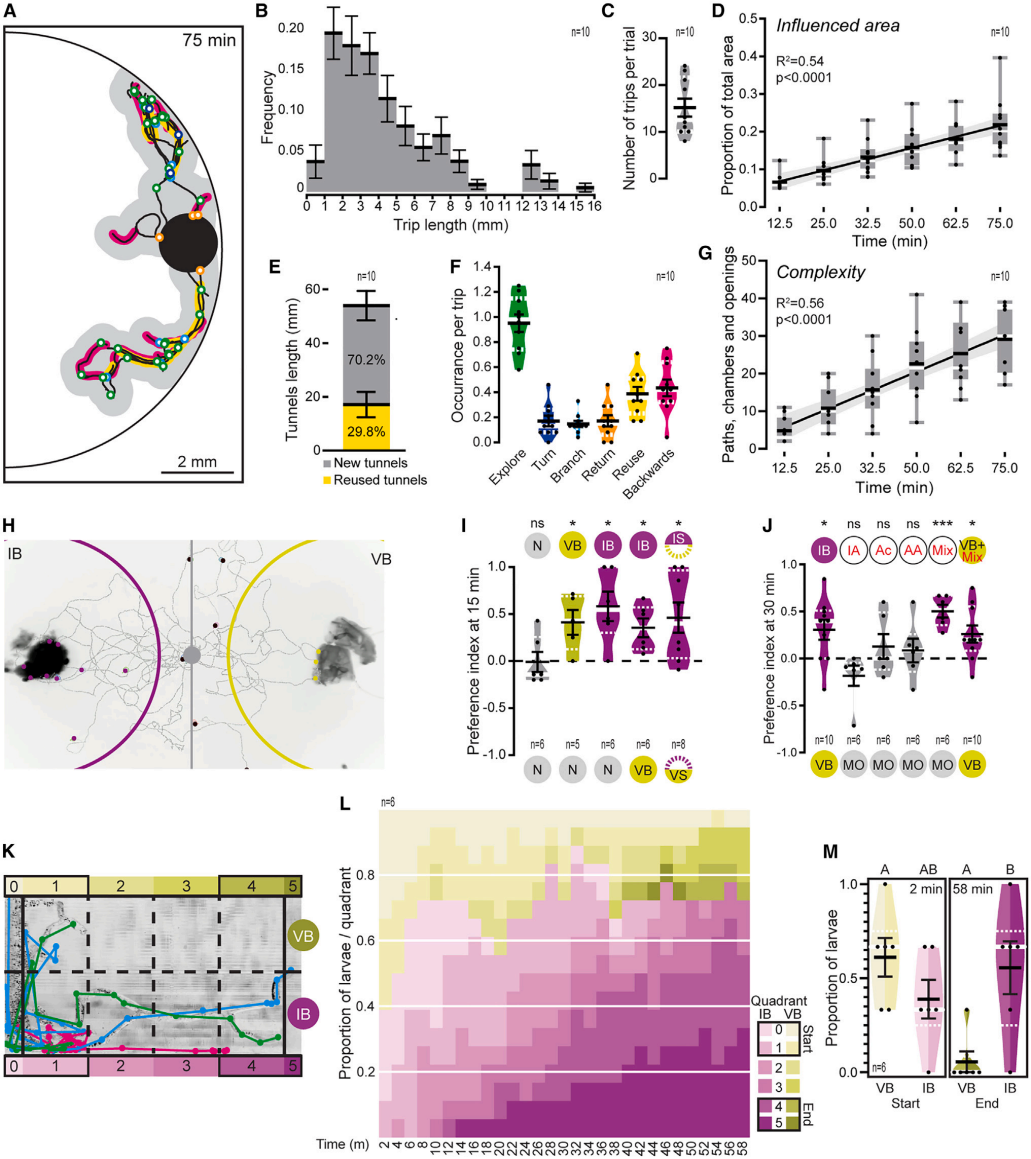
*D. suzukii* larval behavior is conducive to niche construction (A) Example of annotated tracks of a single digging first instar larva after 75 min. Recently hatched larvae were placed in a hole (black circle) in the gel matrix (1% agarose, 10% blueberry juice); yellow: reused tracks; magenta: backward movement; blue: turns; cyan: branching; green: exploration events; orange: returns to the starting point. Gray marks the area considered to have been influenced by larval activity (0.25mm to each side of the track.). (B) Frequency of trip lengths in intervals of 1 mm. Bars represent the mean, error bars the SEM. n represents individual larvae digging trials. (C) Number of trips per trial. White line in the violin plot represents the median, white broken line the 25^th^ and 75^th^ percentiles, black dots the individual values, black line the mean, and error bars the SEM. n represents individual larvae digging trials. (D) Proportion of the total area of the arena that was under the influence of the larva after 75 min in each trial. n represents individual larvae digging trials. Black horizontal lines represent the mean, and error bars the SEM. Boxplots in gray show the 25^th^ and 75^th^ percentiles, whiskers extend to the 10^th^ and 90^th^ percentiles, white line represents the median, and black dots individual data points. Linear regression: regression line (black) with 95% CI (gray). (E) Average length of tunnels from 10 larvae tested individually (n) after 75 min trials, black horizontal lines represent the means, and error bars the SEM. (F) Average number of each behavioral feature occurring per trip. n represents individual larvae digging trials. White line in the violin plots represents the median, white broken line the 25^th^ and 75^th^ percentiles, black dots the individual values, black line the mean, and error bars the SEM. (G) Progression of the complexity of the network of tunnels as quantified by the sum of the number of paths, chambers, and openings to the start point through the 75 min trials. n represents individual larvae digging trials. Black horizontal lines represent the mean, and error bars the SEM. Boxplots in gray show the 25^th^ and 75^th^ percentiles, whiskers extend to the 10^th^ and 90^th^ percentiles, white line represents the median, and black dots individual data points. Linear regression: regression line (black) with 95% CI (gray). (H) Open-field choice assay allowing a choice between larval-infested blueberry (IB, purple perimeter) and virgin blueberry (VB, green perimeter). Larval choice was defined as larval presence within the area around the target tissue. Larvae outside both areas were ignored. Larvae started grouped at the central gray circle. (I and J) Preference index of first instar *D. suzukii* larvae in choice assays at 15 min toward agarose-embedded fruit tissue (I) or at 30 min toward volatile chemicals identified in *D. suzukii* larval modified blueberries (J). N = nothing, VB = virgin blueberry, IB = infested blueberry, VS = virgin side of infested blueberry, IS = infested side of infested blueberry, MO = Mineral oil, IA = Isoamyl alcohol, AA = Acetic acid, Ac = Acetoin, Mix = mixture of the three chemicals, VB + Mix = virgin blueberry additioned with the mix of the three chemicals. White line in the violin plots represents the median, white broken line the 25^th^ and 75^th^ percentiles, black dots the individual values, black line the mean, and error bars the SEM. n represents individual assays with groups of 20 larvae. Individual Student’s *t* tests against a hypothetical mean = 0. (I) N v N, t = 0.08896. N v VB, t = 3.103. N v IB, t = 3.703. VB v IB, t = 3.552. VS v IS, t = 2.862. (J) VB v IB, t = 2.867. MO v IA, t = 1.702. MO v Ac, t = 0.9689. MO v AA, t = 0.6790. MO v Mix, t = 7.486. VB + Mix v VB, t = 2.850. ns: non statistically significant differences, ∗: statistically significant at *p* < 0.05, ∗∗∗: statistically significant at *p* < 0.001. (K) Representative image of a choice digging assay. Traces in green, magenta, and cyan depict the paths taken by the three larvae tested during the assay. Broken lines mark the quadrants defined for quantification. Larvae started the assay on quadrant 0. For the statistical analysis, quadrants 0 and 1 were defined as the Start position, and quadrants 4 and 5 as the End position. Shades of green mark the side of the virgin blueberry tissue, and the green circle, its placement (VB). Shades of purple mark the side of the infested blueberry tissue (IB) and a purple circle its placement. (L) Mean proportion of larvae present in each quadrant at 2 min intervals. Shades of green mark the quadrants on the virgin blueberry side (VB) and purple the quadrants on the infested blueberry side (IB). Black outlines mark the time points analyzed in M. (M) Mean proportion of larvae present at the Start position (quadrants 0–1) at the beginning of the assay (2 min) and at the End position (quadrants 4–5) at the end of the assay (58 min). Binomial generalized linear model (glm) with tissue type and time as explanatory variables different letters indicate statistically significant differences. Explanatory variables: Time (Deviance = −2.209, *p* = 0.137), Side (Deviance = −0.964, *p* = 0.326), Time:Side (Deviance = −13.516, *p* < 0.001). White line in the violin plots represents the median, white broken line the 25^th^ and 75^th^ percentiles, black dots the individual values, black line the mean, and error bars the SEM. n represents individual assays with groups of 3 larvae.

To analyze in detail the behavior of larvae during substrate exploration, we placed first instar larvae in a flat, see-through agarose matrix roughly of the diameter and hardness (Figure S1D) of a fresh blueberry, thin enough to simplify movement to two dimensions (Figures 3A, S2A and S2B). Under these conditions, *D. suzukii* larvae readily dig and travel inside the substrate (Figure 3A). Forward trips within the substrate end in backward crawling along the tunnels to a previous position or in returns to the point of introduction (Figure S2C). The number and length of these forward trips vary substantially, from short bouts of fractions of a millimeter to long journeys of several millimeters (Figures 3B and 3C). In this way, the larvae generate a complex network of tunnels and chambers that radiate from the introduction point, break up the substrate, and bring an increasing area under their influence (Figure 3D). As time goes by, some tunnels are retreaded, and new ones are added to the growing network (Figure 3E). At the same time, as larvae dig and travel through the substrate, they ingest and excrete along the way, seeding the fruit with diffusing excreta (Figure S2D. Video S1). Larval behavior within the substrate is complex, displaying features of exploration and apparent decision-making such as stopping to move the head around and make small indentations to the sides (explore), turning around on the path, branching the tunnels, returning to the origin, and moving backward down the tunnels (Figure 3F). In all, the digging behavior of the larva results in a steady increase in the complexity of the network of tunnels (Figures 3G; S2D). *D. melanogaster* first instar larvae never initiated digging in these assay conditions (data not shown).

Video S1. D. suzukii larval excretion while digging, related to Figure 3

*D. suzukii* larval behavior within the substrate leads to a heterogeneous environment comprised of patches of larva-modified tissue and virgin tissue that have different nutritional qualities. Since larvae seem to explore and choose their paths when they dig, we wondered if they would take advantage of modified tissue and innately seek out and navigate toward these nutritionally richer spots. Therefore, we designed behavioral assays to test the innate preferences of young first instar larvae (Figures 3H and 3K; Figures S3A, S3B, S3F, and S3G).

First, we assayed larval tropism toward both types of tissues. First instar larvae are attracted to both virgin blueberry tissue (VB) and larval-modified infested blueberry tissue (IB) when confronted with no tissue (N vs. VB and N vs. IB, Figure 3I). Interestingly, when given a choice between virgin blueberry tissue and larval-modified infested blueberry tissue, larvae prefer the latter (VB vs. IB). In addition, this holds true even when both types of tissue come from the same fruit (Figure 3I), suggesting that, inside the fruit, larvae would potentially seek out tissue that has been converted into a nutritionally richer substrate by their own activity. On the other hand, *D. melanogaster* larvae are not attracted to virgin blueberry tissue (N vs. VB) but find *D. suzukii* larval infested blueberry tissue attractive (VB vs. IB), which might also explain in part their failure to develop in the former but not the later (Figure S3E). Additionally, we observed that, compared to *D. melanogaster*, *D*. *suzukii* larvae were more solitary and active. They would separate from the initial cluster of larvae much faster, participate more in the assay, and spread and explore a wider area in a shorter amount of time (Figures S3C and S3D).

Having identified characteristic volatile chemicals from *D. suzukii* infested blueberries, we wondered if these odorants would be enough to attract larvae. To determine if *D. suzukii* larvae could detect processed fruit tissue and these volatile chemicals through olfaction, we assayed first instar larvae on a modified two-choice open arena for testing odor-guided behavior (Figure S3B). Larvae find the smell of larval infested fruit tissue attractive and prefer it to the odor of virgin fruit tissue (IB vs. VB, Figure 3J). To our surprise, larvae did not find any of the volatiles present in the *D. suzukii* infested fruit tissue individually attractive (namely acetic acid - AA, acetoin - Ac, or isopentyl alcohol - IA), but they were attracted to a mix of all three volatiles (MO vs. Mix, Figure 3J). Moreover, the addition of this chemical mix to virgin fruit tissue rendered it more attractive than virgin tissue on its own (VB + Mix vs. VB, Figure 3J). Taken together, these results show that larvae are innately attracted to the modified fruit tissue product of their activity in the fruit. These tissues display chemical signs of potential bacterial activity and fermentation. The volatile chemicals found in them are enough to render fruit tissue attractive to *D. suzukii* larvae.

These behavioral experiments on a two-dimensional open field reveal the preferences of the larvae and inform about the senses driving attraction. However, larvae are born in an enclosed environment, where they have to dig their way around to move and feed. We therefore wanted to know if larvae would be able to recognize the presence of different types of fruit tissue, choose, and access them while digging.

To this end, we designed a 3D-printed “behavioral chip” (Figures 3K, S3E and S3F) inspired by previous work on third-instar larvae.^37^ The chip consisted of a chamber filled with agarose with small pieces of both virgin fruit tissue and *D. suzukii* larval-infested fruit tissue embedded on one end and a small space for the larvae to start at the other end. The dimensions of the chamber allowed larvae to move forward, back, and to the sides but not up and down, remaining in a single plane for easier video recording and analysis (Figures S3E and S3F).

We divided the arena into quadrants and quantified the proportion of the total number of larvae found in each at 2.5 min time intervals (Figures 3L and S3F). Focusing on the beginning and the end of the recorded period (1 h), we found that at the onset, larvae were distributed equally among the starting quadrants (VB 0–1 and IB 0–1), while at the end the proportion of larvae found on the larvae-infested fruit end (quadrants IB 4–5) was significantly higher than on the virgin fruit tissue end (quadrants VB 4–5) (Figure 3M). Thus, *D, suzukii* larvae can recognize larval-infested fruit tissue within a gel matrix and readily dig their way to it. Not only that, but also, in this context, they prefer larval-infested fruit tissue to virgin fruit tissue when faced with a choice (Figure 3M).

Interestingly, under these same assay conditions, *D. melanogaster* larvae would never engage in digging. They only crawled around the surface of the agarose matrix and nibbled on it (Video S2). Only when placed together with *D. suzukii* larvae were *D. melanogaster* larvae able to enter the agarose by using the tunnels made by the former (Video S3). *D. suzukii* larvae exhibit from the very early first instar an active digging behavior, not seen so early in development in *D. melanogaster*.

Video S2. Digging assays set up with D. melanogaster larvae, related to Figure 3

Video S3. Coinfestation digging assay, related to Figure 3

## Discussion

In this work, we have studied how *D. suzukii* is able to utilize ripening fruits as a substrate to develop. Our data show that *D. suzukii* larvae, through their activity, provoke changes in the chemical and structural composition of the substrate to sustain their development and, in this way, construct their own niche.

We demonstrate that this process starts from the very onset of larval life as first instar *D. suzukii* larvae make use of their massive mouth hooks (Figure 1C) to overcome the hardness of the substrate and dig through it (Figures 1D and 2A). As infestation progresses, the increasing complexity of the network of tunnels they produce breaks up the tissue and softens it (Figure 3A). The fruit tissue in contact with the larvae increases in protein content and becomes nutritionally richer for the larva (Figure 2B). This richer tissue has a specific chemical signature, a product of *D. suzukii* larval activity, which is attractive to the larvae (Figures 2C and 3J).

The changes in the fruit product of *D. suzukii* infestation are consistent with microbial activity. As they fly and visit feeding and oviposition substrates, *D. melanogaster* and other *Drosophila* species adult flies transport yeasts and bacteria,^38^^,^^39^ possibly promoting the dispersal of microbial species that benefit their development. This could also be the case for *D. suzukii* flies since feeding on specific yeast species has a differential impact on adult fitness^40^ and larval performance.^41^ Although *D. suzukii* adult female flies preferentially lay eggs on ripening fruit with potentially lower microbial load,^11^ they also visit rotting fruits richer in microbes, where they lay eggs^16^ and possibly feed.^42^ Visitations to these types of substrates could serve as sources for microbes that would then be inoculated in the ripening fruit during oviposition.^43^ Interestingly, *D. suzukii* larvae benefit from the growth of specific microbiota, which serve as a source of protein.^44^^,^^45^ The complex tunneling behavior observed in this study (Figures 3A–3G and S2C–S2E) could potentially allow the microbes transported by the adult female during oviposition to enter the fruit tissue and further spread them as feeding continues. Specifically, we show that larvae commonly produce tunnels that return to the point of origin, which in a natural condition would be the oviposition site and possible source of the microbial inoculum. Additionally, as they dig, larvae ingest fruit tissue and excrete in the tunnels, which could further disperse microbes in the interior of the fruit.^39^ Future studies may explore the role of microbes in *D. suzukii*’s niche construction in experiments with axenic flies and sterilized fruits. Furthermore, we found that *D. suzukii*-infested fruits present acetic acid, acetoin, and isoamyl alcohol, which are all produced by microbial fermentation.^32^^,^^33^ Although acetic acid bacteria can be detrimental to *D. suzukii* development through the production of gluconate in certain conditions,^46^ our results show that a mixture of fermentation products including acetic acid are attractive to larvae and may help guide them to microbial growth sites while digging (Figures 2D, 3J and 3K).

In our behavioral assays, larvae showed a preference for the protein-rich larval processed infested fruit tissue over virgin fruit tissue. This is consistent with previous reports indicating that *D. suzukii* larvae prefer and develop better in protein-carbohydrate balanced diets instead of carbohydrate-rich diets^2120^. However, under our semi-natural conditions, we demonstrate that the composition of the environment in which larvae develop is dynamic and patchy, with a temporally and spatially evolving protein content. Noteworthy, while larvae spend the stages of peak mass accrual in their nutritionally enriched medium, first instar *D. suzukii* larvae developing in ripening fruit still have to deal with the nutritionally poor medium it offers. On the contrary, *D. melanogaster* flies are unable to develop past the second instar in ripening fruits but can finish their development in *D. suzukii* larval-infested fruit tissue (Figure 1F). Our results show that for *D. suzukii* larvae, the ripening fruit is a nutritionally poor feeding substrate (Figure 2E), suggesting that first instar *D. suzukii* larvae might present a special resistance to the poor conditions they are born into. Additionally, it has been described that as opposed to *D. melanogaster*, *D. suzukii* third-instar larvae are able to incur in long dives inside the substrate and are more resistant to hypoxia.^47^ It is possible that first instar *D. suzukii* larvae share this resistance. Future studies could explore how *D. suzukii* larval physiology might use nutrients and oxygen differently during the series of larval stages and differently from *D. melanogaster.*

Some behavioral features of *D. suzukii* may provide additional explanations for the differences in performance on the ripening fruit. On the one hand Kim et al.^47^ showed that *D. suzukii* third-instar larvae would dig and dive into much harder substrates that *D. melanogaster*. In our assay conditions, *D. melanogaster* first instar larvae never engaged in digging (Videos S2 and S3), while *D. suzukii* readily did so even engaging in long dives into the substrate. On the other hand, female *D. melanogaster* flies lay eggs in groups, and the larvae aggregate and cooperate to dig and feed more efficiently.^48^^,^^49^^,^^50^^,^^51^^,^^52^ Consistently, in our behavioral experiments, *D. melanogaster* larvae took longer to break out of the initial groups in the center of the plates, and participation in the assays was low (Figures S3C and S3D). Unlike *D. melanogaster*, *D. suzukii* females lay eggs in small clutches of one or a few eggs per fruit.^53^ We observed that first instar *D. suzukii* larvae were very active and readily broke up initial groups, explored the surface and engaged in digging. In all, together with the fact that *D. suzukii* larvae possess massive mouth hooks, our results suggest that in this species, already from early developmental stages, larvae are driven to dig and dive and might not be dependent on aggregation to penetrate the substrate and tunnel through the infested fruit. A genetic basis for larval aggregation behavior has been described in *D. melanogaster*.^50^^,^^54^ It would be interesting to investigate how these genes or their expression levels have diverged in *D. suzukii*.

Furthermore, differences in oviposition site preference were observed between the two species, with *D. melanogaster* favoring soft substrates and *D. suzukii*, hard substrates. The underlying mechanosensory receptors and neurons involved in both species have been partially described.^15^^,^^55^^,^^56^ Interestingly, a previous study has suggested that the evolution of *D. suzukii*’s capacity to exploit the hard ripening fruit as an oviposition niche is supported by a co-evolution of changes in ovipositor structure and modification of multiple sensory modalities.^15^ In this sense, future studies could explore the evolutionary history of the mouth hook structure and the associated mechanosensory system in related species to investigate whether these and the genes controlling their development and function have co-evolved.

Based on our observations, we propose that *D. suzukii* larvae are able to develop in their mother’s preferred oviposition niche by modifying it according to their needs. Many animals can occupy niches that would otherwise be inhospitable by modifying an existing environment through their activity, thus constructing their niche.^57^^,^^58^ Earthworms produce complex tunnels that mix the soil and change its properties,^59^ allowing their survival and the establishment of whole ecosystems. Bark beetles bore into tree trunks, introducing fungi, with which they shape the otherwise inaccessible and lethal internal environment of the trees, and Ambrosia beetles have even evolved true fungi farming.^60^^,^^61^^,^^62^ Moreover, it has been proposed that yeasts construct their niche in rotten fruit, facilitating a mutualistic relationship with *Drosophila* flies.^17^^,^^38^^,^^39^ As for the case of *D. suzukii*, we propose that larvae are physically and behaviorally adapted to dig in hard fruit substrate from the very beginning of their life, possibly allowing the introduction of beneficial microbes in the fruit, which will grow in the fruit and serve as an additional nutrient source for the later stages of larval development. In this way, *D. suzukii* larvae modify the environment and construct their own niche. Many studies have already addressed the fitness consequences of yeasts and bacteria on adult and larval life history traits in *D. suzukii*^6^^,^^40^^,^^41^^,^^45^^,^^46^, but an analysis considering the niche construction perspective is still missing. Since the phenomenon of niche construction can potentially lead to shifts in selective pressures and constitute a driver for the evolution of the organisms involved,^63^ future studies could make use of the methods to work on a natural substrate we developed, to address whether and which microorganisms are involved and their fitness consequences and relationships.

### Limitations of the study

Our study comes with some limitations, mainly arising from a need to reduce the number of variables while trying to simulate natural conditions. First, the use of strains maintained in laboratory conditions could potentially impact behavioral responses and fly development and survival, as well as possibly have a different associated microbiota compared to flies raised in their natural environment. For example, *D. melanogaster* sensitivity to substrate texture depends on genetic background.^64^ In addition, differences in the rearing conditions for *D. melanogaster* and *D. suzukii* stocks to conform to the particular needs of each species could potentially result in different microorganisms being associated with each stock and carried over in the experiments.

Here, we approximate natural *D. suzukii* infestation conditions by performing experiments directly on fruits isolated from their plants, and we cannot rule out different compositional changes in isolated and plant-attached fruits. Furthermore, contrary to our experiments, in natural field conditions, the quantity of larvae per fruit is likely variable. Regardless, we consider that the procedures we developed are a valuable contribution to the future study of this species in relation to its host plants.

Finally, in this study, we limited the analysis to blueberries from commercial sources, and the dynamics we found may differ in other host fruits from other origins. *D. suzukii* finds home in a wide variety of fruits of economic importance.^65^^,^^66^ These fruits differ in size, color, firmness, and organoleptic qualities. They are thus likely to have a different nutritional composition and microbiota living on them. Furthermore, adult female flies show a differential preference for these different fruits, and larvae performance on them is variable^22^^,^^25^; therefore, future studies may want to analyze how *D. suzukii* activity converts other fruit hosts.

## Resource availability

### Lead contact

Requests for resources, instructions and data should be directed to and will be fulfilled by the lead contact, B.S.H. (hansson@ice.mpg.de).

### Materials availability

Further instructions to build the behavioral setups we developed are available upon request to the lead contact.

### Data and code availability

• Data: All data reported in this paper will be shared by the lead contact upon request.
• Code: This paper does not report original code.
• Additional information: Any additional information required to reanalyze the data reported in this paper is available from the lead contact upon request.

## Acknowledgments

We thank the European Commission and the Marie-Skłodowska Curie Actions (MSCA-IF-EF-CAR-838205), and the Max Planck Society for financing the study. We would like to thank Dr. Pierre Leopold for kindly sharing with us the dILP antibodies. We thank the technical assistance of Angela Lehmann, Roland Spiess, Jasmin Grosse, Daniel Veit, Veit Grabe, Silke Trautheim, and Regina Stieber, as well as the service and administration sections of the Max Planck Institute for Chemical Ecology.

## Author contributions

Conceptualization, D.G.; Methodology, D.G. and A.D.C.; Formal Analysis, D.G., G.K., and A.D.C.; Investigation, D.G. and A.D.C.; Writing – Original Draft, D.G. and A.D.C.; Writing – Review and Editing, D.G., A.D.C., G.K., M.K., and B.S.H.; Visualization, D.G.; Supervision, M.K. and B.S.H.; Project Administration, M.K. and B.S.H.; Funding Acquisition, D.G., M.K., and B.S.H.

## Declaration of interests

The authors declare no competing interests.

## STAR★Methods

### Key resources table

**Table:** 

REAGENT or RESOURCE	SOURCE	IDENTIFIER
**Antibodies**		

Rabbit anti-Dilp2	Géminard et al.^34^	RRID:AB_2314316

**Critical commercial assays**		

Pierce Rapid Gold BCA Protein Assay Kit	Thermo Scientific	Cat#A53225
SPME Fiber Assembly	Sigma-Aldrich	Cat#57328-U

**Experimental models: Organisms/strains**		

*Drosophila suzukii*	National Drosophila Species Stock Center	14023–0311.03
*Drosophila virilis*	National Drosophila Species Stock Center	15010–1051.00
*Drosophila biarmipes*	National Drosophila Species Stock Center	14023–0361.10
*Drosophila subpulchrella*	EHIME-Fly srock center	E−15203
*Drosophila melanogaster* Canton-S	Bloomington Drosophila Stock Center	64349
*Drosophila suzukii* Orco-Gal4>UAS-GCamp6f	Depetris-Chauvin et al.^67^	N/A

**Software and algorithms**		

Fiji	Schindelin et al.^68^	N/A
ZEN Blue microscopy software	Zeiss Microscopy	N/A
MSD ChemStation F.01.03.2357	Agilent	N/A
Mass Spectral Search Software 2.2	National Institute of Standards and Technology	N/A
BioImageOperation Software	Cano-Ferrer et al.^69^	N/A
R version 4.2.3	R Core Team^70^	N/A
GraphPad Prism 9	GraphPad Software	N/A
InfoStat package version 2009	Grupo InfoStat, FCA, Universidad Nacional de Cordoba, Argentina	N/A

**Other**		

Nylon mesh biopsy bags	Epredia	Cat#6774011

### Experimental model and study participant details

*D. suzukii* wild-type (14023–0311.03), *D. virilis* (15010–1051.00), and *D. biarmipes* (14023–0361.10) strains were obtained from National Drosophila Species Stock Center. *D. subpulchrella* was obtained from the EHIME-Fly srock center (E−15203). These stocks were maintained at 23°C in optimized fly-rearing medium vials. For *D. suzukii*, *D. subpulchrella,* and *D. biarmipes* vials were supplemented with crushed blueberries. A piece of crumpled tissue paper was inserted into the vials to allow flies to perch. *D. melanogaster* Canton-S were obtained from the Bloomington Drosophila Stock Center (64349) and maintained at 25°C in optimized fly-rearing medium vials. *D. suzukii* Orco-Gal4>UAS-GCamp6f^67^ were used as a marked strain to enable discrimination from wild-type flies in concurrent infestation experiments; these were maintained in the same conditions as the wild-type strain.

### Method details

#### Larval development analysis

To follow larval development inside blueberries, we allowed flies to lay eggs on agar plates for 6 h. For *D. suzukii* flies, we used 3% agar, 10% blueberry juice, 2% strawberry syrup plates supplemented with dead yeast, potato starch, and strawberry syrup paste. For *D. melanogaster,* we used 1.5% agar, 3% sucrose plates supplemented with 1% v/v 30% Nipagin in ethanol, supplemented with a live baker yeast paste. After oviposition, yeast was removed from the plates, and embryos were allowed to develop for 24 h at 25°C. Blueberries were obtained from local supermarkets.

To place larvae inside the fruits, a V-shaped slit was made on the skin of the blueberries, and a skin flap was lifted. Five larvae were picked form the plates and placed directly on the exposed fruit flesh. The skin flap was closed and individual fruits were placed in individual nylon mesh biopsy bags (Epredia - 6774011) closed with twist ties. Fruits were hung inside an incubator at 23°C, 70% humidity, and a 12h light-dark cycle. For co-infestation experiments, five larvae of each species were placed simultaneously on each fruit. To analyze *D. melanogaster* development on softened fruit, fruits were softened by lightly pressing them with the fingers.

To monitor developmental timing, blueberries were removed from the incubator at the reported time points, opened with tweezers and scalpels, and searched for larvae under a Leica S8AP0 stereomicroscope. Developmental stages were scored according to mouth hook and spiracle appearance. Larval weight was measured using a Sartorius ME235p precision scale. Day 0 larvae were weighed in groups of 20, day 1 larvae in groups of 10, day 3 larvae in groups of 2, and from day 4 onward larvae were weighed individually.

To monitor developmental timing of *D. melanogaster* in optimized fly rearing medium, 5 recently hatched first instar larvae were placed in 15 mL Falcon tubes cut at the 6mL mark, filled with 2 mL of rearing medium and closed with foam stoppers. The tubes have a diameter of 1.4 cm, which can approximate a large blueberry. Tubes with larvae were kept at 25°C, 70% humidity, and 12h light-dark cycle. Developmental stages were scored according to mouth hook and spiracle appearance.

To assess survival and development of *D. melanogaster* larvae in *D. suzukii* larva-modified blueberry tissue, we removed the tissue that presented signs of larval activity (presence of larvae, darkening, softness) from 4-day post-*D. suzukii* infestation blueberries maintained in the aforementioned conditions. We placed individual tissue samples in 2 mL Eppendorf tubes with their caps removed to a volume of around 500 mL. We placed 5 recently hatched first instar *D. melanogaster* larvae in each tube and closed the tubes with foam stoppers. We maintained these tubes in a plastic rack with a loosely fitting transparent plastic lid at 25°C, 70% humidity, and 12h light-dark cycle and scored the number of pupae and adult flies once a day.

#### Larval mouth hook imaging and measurement

To image larval mouth hooks, larvae were treated using a simple clearing technique.^71^ Recently hatched first instar larvae were collected from oviposition plates and placed in a 1.5 mL Eppendorff tube, and 1 mL of boiling hot water was added while the tube was also placed in a boiling hot water bath for a few minutes; this kills the larvae instantly and avoids shrinkage and browning. Afterward, water was carefully removed and replaced with 70% ethanol. After 30 min incubation this was replaced by 100% ethanol, incubated for 30 min, and replaced with methyl salicylate. Clearance of larval tissues is immediate; they become invisible in suspension, and their presence can be gauged only by the floating mouth hooks. Larvae were mounted on a glass slide without changing the medium. Slides were imaged by differential interference contrast microscopy with a Zeiss Imager Z1 microscope. Measurements were made with Fiji software^68^ using *D. melanogaster* third instar larva cephaloskeleton description^72^ as a reference to determine landmarks.

#### Media hardness measurements

We measured the hardness of different media by assessing the peak compressive force required to penetrate 5 mm into the media using a Sauter FH50 force gauge fitted with a conical tip part of the AC43 standard attachment pack for the apparatus. Agarose gels and fly rearing medium were made in 2 mL volume, 1.5 cm diameter cylindrical cups. Fresh and softened blueberries were carefully pealed under a steromicroscope to reveal the flesh before measurements.

#### Blueberry flesh color measurements

To measure the color intensity of blueberry flesh infested by *D. suzukii* or *D. melanogaster*, we bisected 3 and 4-day post-infestation fruits with a scalpel longitudinally along the infestation site. We placed a few drops of water on the exposed fruit interior and covered it with a microscope cover glass. We imaged the fruits in a Zeiss Axio Zoom V.16 stereomicroscope fitted with a CL 9000 LED ring illuminator, an Axio 506 color camera, and a PlanApo Z 0.5x/0.125 FWD 114 mm objective. We imaged the fruits at 30% illumination intensity, 10x magnification, and 280 ms exposure time, setting the white balance with a ColorChecker Classic Mini color reference card (Calibrite) using ZEN Blue microscopy software (Zeiss Microscopy). From the pictures taken, we split the red, green, and blue channels and measured mean gray intensities in each channel in circular regions of interest of equal area on each picture using Fiji software.^68^

#### Protein quantification

To extract proteins from fruit tissue for measurements, infested fruits were cut in half manually transversally to the point of infestation at different time points of the larval development. Under a Leica stereomicroscope, tissue displaying signs of larval activity (softer and darker tissue, and tissue where larvae were found) and virgin fruit tissue were carefully removed with scalpels, using individual clean tools for each type of tissue. All larvae from the samples were removed. Approximately 120–140 mg of fruit tissue per sample was weighed on a Sartorius ME235p precision scale, recording the exact weight for each sample. Maintaining samples on ice, we placed them in Precellys Tissue Homogenizing CKMix tubes (P000918-LYSK0-A – Bertin Technologies) and froze them by submerging the tubes in liquid nitrogen. We then added 1.5 mL water supplemented with Halt Protease Inhibitor Cocktail (87786 – Thermo Scientific) and processed them for 30 s at 50 rpm on a Quiagen Tissueliser LT (Quiagen). We centrifuged the samples at 10000 g for 5 min at 4°C, recovered 1 mL of supernatant, and stored it at −20°C until measurement.

To measure the protein content, we made 1:5 dilutions of these extracts in water and used a Pierce Rapid Gold BCA Protein Assay Kit (A53225 – Thermo Scientific) proceeding according to manufacturer’s instructions using an Infinite M Nano+ plate reader (TECAN). We determined protein amount by comparing to BSA standard curves ran on each sample plate and normalizing by the measured weight of each original tissue sample.

#### Fruit chemical analysis by solid-phase microextraction

Fruit volatiles were collected by solid-phase microextraction (SPME). Briefly, blueberries with different treatments (sham, *D.melanogaster*, or *D. suzukii* infested) and at different time points after infestation (1, 3, 4, 5, and 7 days) were cut in half, placed individually in N18 20 mL glass vials, and closed with a cap equipped with a polytetra-fluoroethylene-lined silicone septum (Sigma-Aldrich, 23242-U). An SPME fiber (gray fiber coated with 50/30-μm divinylbenzene/carboxen on polydimethylsiloxane on a StableFlex fiber, Sigma-Aldrich, 57328-U) was inserted through the silicone septum and exposed to the fruit headspace for 10 min at room temperature. The SPME fiber was then retracted and immediately inserted into the GC-MS system supplied with an HP-INNOWAX column (Agilent 19091*N*-133UI). Samples were injected at an initial oven temperature of 50°C, held for 2 min, and increased gradually (15 °C min^−1^) to 250°C and held for 5 min. The mass spectrometry transfer line was held at 280°C, the mass spectrometry source at 230°C, and the mass spectrometry quad at 150°C. Mass spectra were taken in EI-mode (70 eV) in a 33–350 m/z range. All chromatograms were analyzed using MSD ChemStation F.01.03.2357 software (Agilent), and chemicals were identified using the NIST library (NIST Mass Spectral Search Software 2.2) and matched to the Max Planck Institute for Chemical Ecology library standards.

#### Insulin like peptide quantification

To assess the influence of fruit processing by *D. suzukii* larvae on the perceived nutritional quality of the substrate, we analyzed the levels of dILP2 in the IPCs of *D. melanogaster* and *D. suzukii* early third-instar larvae fed 24 h on *D. suzukii* processed blueberries.

We collected recently hatched first instar *D. suzukii* Orco-Gal4>UAS-GCamp6f larvae and infested blueberries with five of them per fruit. We allowed these larvae to develop for five days at 23°C, which corresponds to our observed period of increased in protein content in the fruit and when larvae have reached the third instar. In a staggered manner in time we separately infested blueberries with wild-type *D. suzukii* and allowed them to develop for four days at 23°C, until they reached the early third instar. Similarly, we placed first instar *D. melanogaster* larvae on oviposition plates covered with yeast paste and allowed them to develop for three days at 25°C until they reached the early third instar. We removed the early third instar wild-type larvae from their development media, thoroughly rinsed them with water and transferred groups of five of each species to individual fruits infested with the transgenic *D. suzukii* larvae on their fifth day of development. Simultaneously, we also transferred groups of five wild-type larvae of each species to virgin blueberries. We allowed all to develop for 24 h at 23°C.

We then removed all larvae from the fruits and sorted them under a fluorescence stereomicroscope to recover the wild-type larvae. The transgenic flies used to infest the blueberries express fluorescent proteins that helped us distinguish them from the wild-type flies in the co-infestation context. We rinsed the larvae thoroughly and proceeded to immunostaining and fluorescence imaging.

We dissected the larvae in PBS, cutting their head, inverting it, and removing the fat body and the gut, to expose the brain still attached to the cuticle. We fixed in Paraformaldehyde 4% for 45 min and rinsed 3 times for 15 min with PBS, 0.3% Triton X-100. We then blocked with 5% Normal Goat Serum (Merck - #S26-100ML) in PBS, 0.3% Triton X-100. Afterward, we incubated with rat anti-DILP2 antibody 1:100^34^ overnight at 4°C. We then washed 3 times for 15 min with PBS, 0.3% Triton X-100, and incubated with goat anti-rat Alexa 488 50% glycerol secondary antibody 1:125 (Thermo Fisher - #A-11006) in PBS, 0.3% Triton X-100 for 2 h. We then washed 3 times for 15 min with PBS, 0.3% Triton X-100, dissected the brains from the cuticles, and mounted them on microscope slides with Vectashield mounting medium (H-2000 – Vector Laboratories). We imaged the samples on a Zeiss LSM 880 Confocal microscope. All pictures were taken employing the same confocal settings. For Dilp2 immunoreactive analysis within the IPCs, confocal images of brains stained against Dilp2 were taken with a 40X objective and an optical zoom of 3 using a 0.69 mm step size. First, a Z projection of 6 stacks showing Dilp2 signal was made. Then, a region of interest (ROI) was selected, adjusting the threshold image in order to mark most of the Dilp2 signal. Mean Dilp2 fluorescence intensity and area were measured within the ROI created. A rectangle of the same or a higher area was located outside of the IPCs and used to subtract the background signal. For the analysis, we considered the mean gray value (with the subtracted background signal) a measurement that is independent of the area. Data was normalized to the average dilp2 signal of each independent experiment to compare between species. Quantification was performed with Fiji software.^68^

#### Larval behavior

In all assays individual first instar larvae were picked from plates 24 to 28 h after oviposition.

#### Free digging assay

An apparatus to observe larvae freely digging was produced from laser cut 3 mm transparent Plexiglas. The apparatus consisted of two pieces (Figure S2B) fit together with stainless steel bolts and nuts and brass washers (Figure S2A). A solution of 1% agarose and 10% blueberry juice was boiled and applied while still hot in the 12 mm well of the bottom piece and immediately covered with the top pieces. Bolts and nuts were fastened, and agarose was let to gel. The top piece had two holes that could be used as the starting spot for the larvae once agarose was removed from them. The starting position was placed to the left or right side alternatively on each repetition of the assay. One larva was assayed per replicate.

#### Visualization of larval excretion

Recently hatched first instar *D. suzukii* larvae were placed for 1 h on 1% agarose plates supplemented with sulphorohdamine. Afterward, they were washed and placed in the free-digging assay apparatus and recorded with a Zeiss Axio Zoom V.16 fluorescence stereomicroscope.

#### Digging choice assays

An apparatus to observe larval digging behavior consisting of a 3D printed piece and a 3 mm transparent laser cut Plexiglas cover was produced (Figure S3F). Pieces of fruit tissue to serve as goals for the larvae were placed in the chamber, and the two pieces of the apparatus were fit together with stainless steel bolts and nuts, and brass washers (Figure S3F). A solution of 1% agarose was boiled and applied while still hot through the square loading hole (Figure S3F) of the assembled apparatus. Agarose plugging the loading hole was removed before the assay to allow larvae to be loaded. Once larvae were placed, the hole was plugged with a neutral molding paste. The position of the goals (top or bottom) was switched on each repetition of the assay. Three first instar larvae were placed in the assay apparatus per replicate. To allow recording, assays were conducted with white light illumination.

#### Open field choice assays

To image open field choice assays, we used frustrated total internal reflection.^73^ We built an apparatus consisting of a 10 cm in diameter and 8 mm thick transparent acrylic base illuminated by infrared LEDs placed around it. The base was placed on a frame that supported an infrared-sensitive camera above it. The assay was conducted with the infrared LEDs as the only illumination source. On top of the base an 8 cm diameter circular 1% agarose gel was placed. The gel was made by pouring 13 mL hot agarose solution in disposable 9 cm diameter culture plates to a thickness of around 0.5 cm. Larvae were placed in groups of 20 per replicate in the center of the gel. Goals for the larvae were placed 1.5 cm away from the edge of the agarose. To embed tissues in the agarose, fruit tissue samples were placed on the positions in the empty culture plate before agarose was poured so that after gelling samples remained embedded (Figure S3A). In the cases where goals (chemical solutions or tissue samples) were isolated from the agar, these were placed in inverted 1.5 mL Eppendorf tube caps used as containers on top of the gelled agarose (Figure S3B) immediately before the assay. The position of the goals was switched on each repetition of the assay.

#### Video recording and processing

An Alvium 1800 U-158m camera (Allied Vision, Inc - #14184) was used to record all videos. In digging assays it was fitted to a Leica S8AP0 stereomicroscope with an Olympus KL200 illuminator. Videos were streamed to a computer using Vimba Viewer software (Allied Vision Technologies), and recorded with OBS studio (https://obsproject.com/).

Videos were processed with BioImageOperation software.^69^ Frames were extracted to match the required time points for analysis. In open field choice assays, individual larvae were automatically tracked, and the software generated videos with the marked tracks. Videos were annotated manually using Adobe Illustrator (Adobe Systems Incorporated). The complexity of the tunnel system was quantified as the sum of paths, chambers and openings to the staring point.^74^ The co-infestation video (Video S2) was annotated manually with Bio-Image Indexing and Graphical Labeling Environment (BIIGLE).^75^ Quantification of larval influence area was done with Fiji.^68^

### Quantification and statistical analysis

Here we detail the statistical methods used in each figure were data were compared. Number of replicates and their definitions can be found in each figure legend. When not stated otherwise, data were analyzed using R version 4.2.3.^75^

Figure 1B. To test whether larvae of the two *Drosophila* species differ in their survival a binomial generalized linear model (glm) was used. Slight overdispersion was detected and corrected using a quasi-glm model. P-values were obtained by comparing models with and without the explanatory variable with an analysis of deviance.^76^ The number of replicates (n), means and SEM can be found in the figure. Statistical values (F and *p* values) can be found in the figure legend.

Figure 1C. Morphological data from the two *Drosophila* species were compared with ANOVA. The number of replicates (n), means and SEM can be found in the figure. Statistical values (F and *p* values) can be found in the figure legend.

Figure 2B. The influence of the presence of *D. suzukii* and time (both used as categorical explanatory variables) on the protein content was investigated using the generalized least squares method (gls from the nlme library^77^^,^^78^) to account for the variance heterogeneity of the residuals. The data were log-transformed and the varIdent variance structure was used. Whether the different variance of treatment (presence of *D. suzukii*), time, or the combination of both factors should be incorporated into the model, was determined by comparing models with different variance structures with a likelihood ratio test and choosing the model with the smallest AIC. The influence (*p*-values) of the explanatory variables was determined by sequential removal of explanatory variables from starting from the full model, and comparison of the simpler with the more complex model with a likelihood ratio test.^76^ Differences between factor levels were determined by factor level reduction.^79^ The number of replicates (n), means and SEM, as well as the statistical values (likelihood ratios and *p* values) can be found in the figure.

Figure 2C. The larval weight followed a 3-parameter sigmoidal growth curve. This was calculated using the self-starting nonlinear least squares (nLs) logistic model (SSlogis) which follows the form:

$$\frac{a}{1+e^{-\left(\frac{x-b}{c}\right)}}$$

Number of replicates, measured means and SEM as well as estimated parameters of the growth curve and their standard errors are shown in the graph. Statistical values (t and *p* values) are given in the figure legend.

Figure 2E. The immunofluorescence data were analyzed using a two-way ANOVA with tissue type and *Drosophila* species as explanatory variables. In order to achieve normality of the residuals and variance homogeneity data were square root transformed. The number of replicates (n), the mean dILP2 signals as well as the SEM are given in the graph. Statistical values (F and *p* values) are given in the figure legend.

Figures 3D and 3G. The increase of the influence area and complexity of the tunnel system (this was calculated as the sum of the number of chambers, paths and openings to the starting point^74^) during larval digging were anaylized by linear regression using GraphPad Prism 9 (GraphPad Software). The number of replicates (n), the mean area and complexity values as well as the SEM for each time point are given in the graphs. Statistical values (R^2^ and *p* values) are given in the graphs.

Figures 3I, 3J, and S3E. To determine whether larvae presented preferences in choice assays, we calculated a preference index as the total of larvae in the target area/total of larvae that participated in the assay (exited the central spot were they were originally placed). We analyzed data with Student-t Tests against a hypothetical mean = 0 (absence of preference) using GraphPad Prism 9 (GraphPad Software). The number of replicates (n), the mean preference indeces and SEM are given in the graphs. Individual values, the median, the 25^th^ and 75^th^ percentile are also given in the graphs. The statistical values (t and *p* values) are given in the figure legend.

Figure 3M. To test whether larvae of *D. suzukii* differ in their choice a binomial generalized linear model (glm) with tissue type and time as explanatory variables was used. *p*-values were obtained by sequencially removing explanatory variables and comparing the more complex model with the next simpler model with the analysis of deviance.^76^ In order to find out which groups differed from each other the TukeyHSD test was applied using the package ‘emmeans’.^80^ Average proportions of larvae and number of replicates (n) are given in the graph. Statistical values can be found in the figure legend.

Figure S1B. To compare the weight of first instar *D. suzukii* and *D. melanogaster* larvae we used a Student-t Test with GraphPad Prism 9 (GraphPad Software). Individual values, median, 25^th^ and 75^th^ percentiles, and number of replicates (n) are given in the graph. and number of replicates (n) are given in the graph. Statistical values can be found in the figure legend.

Figure S1C. To compare the hardness of the different media we tested the ANOVA requirements and our data did not fulfill normality and variance homogeneity criteria, so we performed a Kruskal-Wallis non-parametric test InfoStat package version 2009 (Grupo InfoStat, FCA, Universidad Nacional de Cordoba, Argentina). Individual values, median, 25^th^ and 75^th^ percentiles, mean, SEM, and number of replicates (n) are given in the graph. Statistical values can be found in the figure legend.C.

Figure S1E. To test the changed in color that occur within fruits after infestation, mean gray values of each RGB color channel were analyzed with linear mixed effects models using the lmer function of the lmerTest package.^81^ Individual fruits were treated as random effects (random intercept), and side of the fruit and treatment as fixed effects. To achive variance homogeneity color values were log-transformed. *p* values for explanatory variables were obtained by deleting explanatory variables one after another and comparing the the more complex with the simpler model.^76^ Mean intensity values ± SEM, number of replicates as well as statistical values can be found in the figure.

Figure S3D. To compare the movement of *D. melanogaster* and *D. suzukii* first instar larvae, we compared the proportion of larvae which were in a certain area in the arena with a binomial generalized linear mixed models (binomial glmm) using the glmer function of the lme4 package^82^ to account for repeated observations. Replicates were treated as random effects (random intercept), and time of the observation and *Drosophila* species as fixed effects. *p* values for explanatory variables were obtained by deleting explanatory variables one after another and comparing the the more complex with the simpler model.^76^ Mean proportions ± SEM, number of replicates (n) as well as statistical values can be found in the figure.
