# Supplementary material for: Shaping the environment – Drosophila suzukii larvae construct their own niche
Source: iScience. 2024 Nov 8;27(12):111341. doi: 10.1016/j.isci.2024.111341 (PMC11647167; doi:10.1016/j.isci.2024.111341)
Supplement: Document S1. Figures S1–S3 [file mmc1.pdf]

iScience, Volume *27*

Supplemental information

Shaping the environment – *Drosophila suzukii* larvae construct their own niche

Diego Galagovsky, Ana Depetris-Chauvin, Grit Kunert, Markus Knaden, and Bill S. Hansson

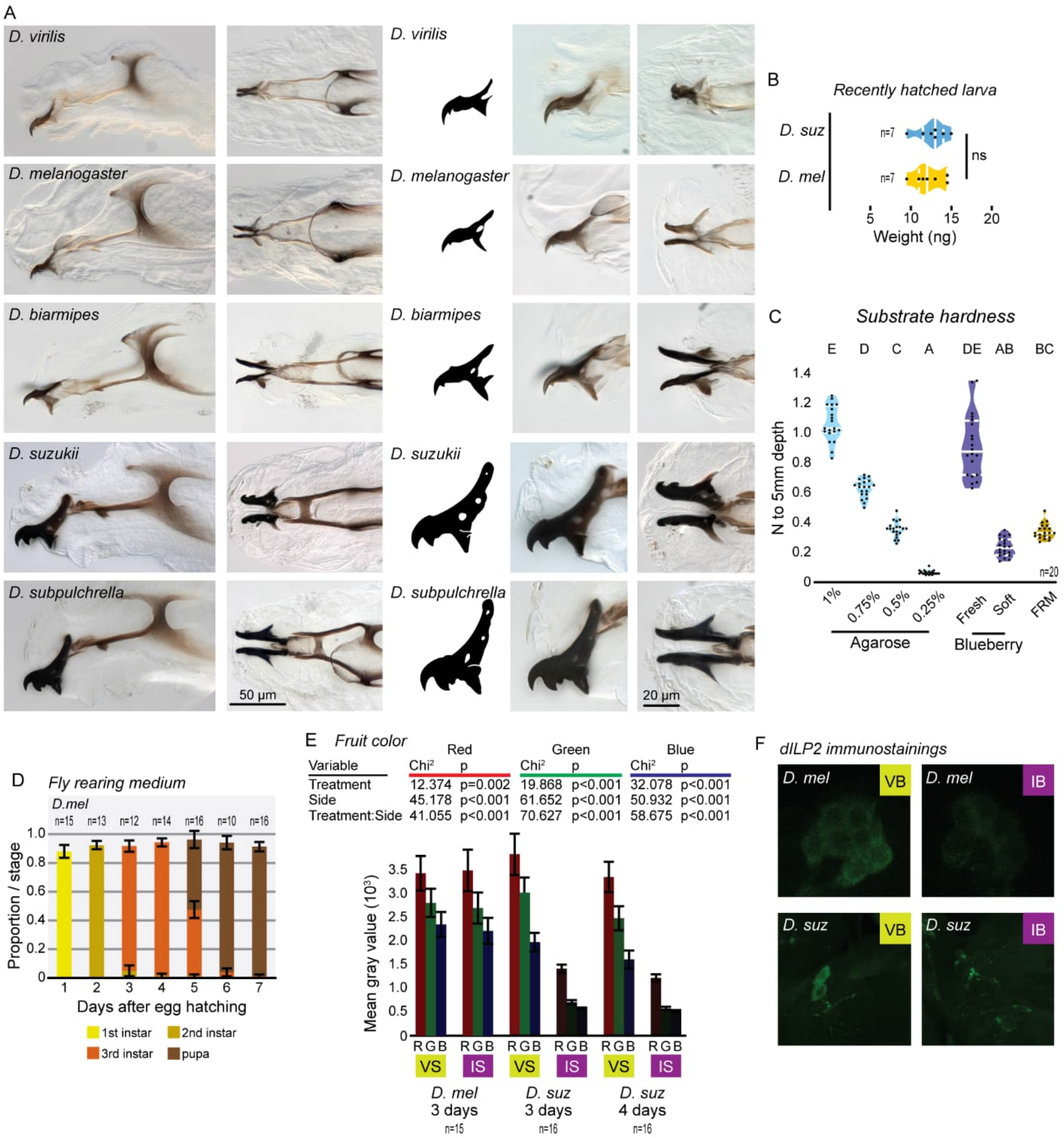

*Figure S1. First instar larval mouth hooks, **larval development and fruit tissue modification**, related to Figures 1 and 2. A. Pictures showing a side view (first and third columns) and a bottom view (second and fourth columns) of the mouth hooks of D. virilis, D. melanogaster, D. biarmipes, D. suzukii and D. subpulchrella. In the central column, enlarged isolated tracings of the structures are shown for a clearer comparison. B. Weight of individual recently hatched first instar larvae of D. suzukii and D. melanogaster (ng). Values of individual larvae were estimated from weighed groups of 20 larvae. Black dots: individual values. White horizontal bar: median. Dotted white lines: 25th and 75th percentiles. Student-t Test. t = 0.3693. ns: non statistically significant differences, n: number of measured groups of larvae. C. Compressive resistance (N to 5mm) as a measure of the hardness of Agarose 1%, 0.75%, 0.5% and 0.25%*

in water with 10% blueberry juice, fresh blueberries, manually-softened blueberries (Soft) and fly rearing medium (FRM). Black dots, individual values. White horizontal bar, median. Dotted white lines, 25th and 75th percentiles. Kruskal-Wallis non-parametric test. H = 129.07, p < 0.0001, n: number of tested substrate samples. Same letters indicate non-statistically significant differences. D. Average proportion of the initially placed first instar larvae of D. melanogaster in each developmental stage, found in each vial of fly rearing medium at one-day intervals after introduction (days after egg hatching). Bars indicate the mean, error bars the SEM, n the number of investigated vials. E. Measurement of intensity values (mean gray values) in the Red (R), Green (G), and Blue (B) channels of pictures of blueberries 3 days post infestation with D. melanogaster and 3 and 4 days post infestation on the virgin side of the fruit (VS) and the infested side of the fruit (IS). To illustrate the differences, the columns are colored with 2x value of the measured average intensity of the corresponding channel. Color values were analyzed with linear mixed effects models (see Materials and Methods for details)*. Bars represent the mean, error bars the SEM and n the number of fruits measured. F. Representative immunostainings against dILP2 in the IPCs of third instar D. melanogaster (left) and D. suzukii (right), fed on virgin blueberry (VB) of D. suzukii infested blueberry (IB).*

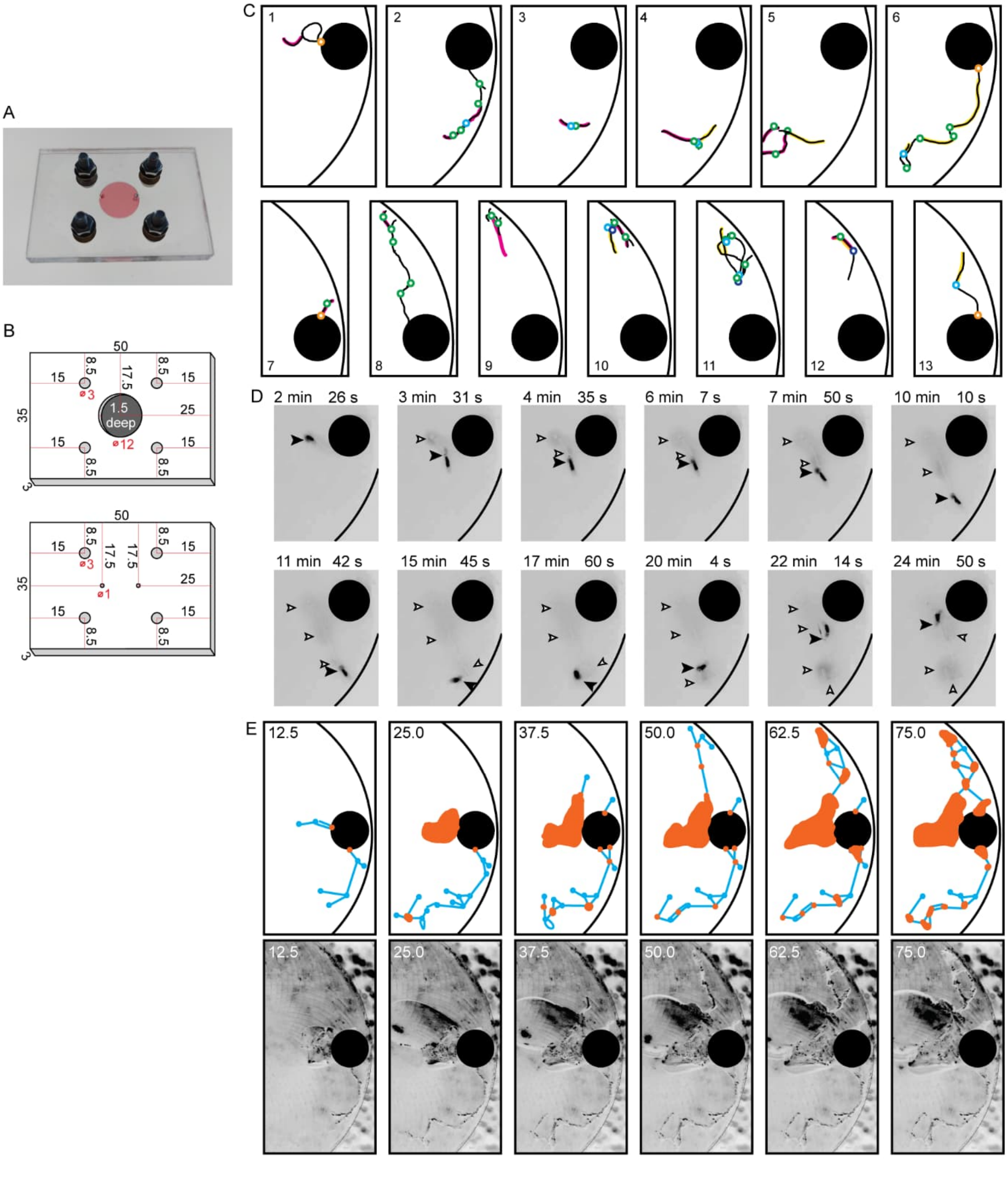

Figure S2. Free digging assays, related to Figure 3. A. Picture of the assembled apparatus. B. Schematics of the apparatus, sizes expressed in mm. C. The thirteen individual trips (defined as forward locomotion ending in backward movement or return to the starting point) that make up the tracks from the example presented in Figure 3A. Yellow: reused tracks; magenta: backward movement; blue: turns; cyan: branching; green: exploration events; orange: returns to the starting point; black circle: starting point. D. First instar D. suzukii larva previously fed on sulforhodamine colored agarose digging through uncolored agarose. The selected frames correspond to moments when excretion occurred (solid arrowheads). Clear arrowheads

mark diffusing excreta from previous frames. E. Complexity analysis of the example repetition of the assay from Figure 3A. On the top row, orange marks the chambers and the openings to the starting point identified at each time point, and blue marks the individual paths connecting them. The bottom row shows the extracted frames from the video used for the analysis.

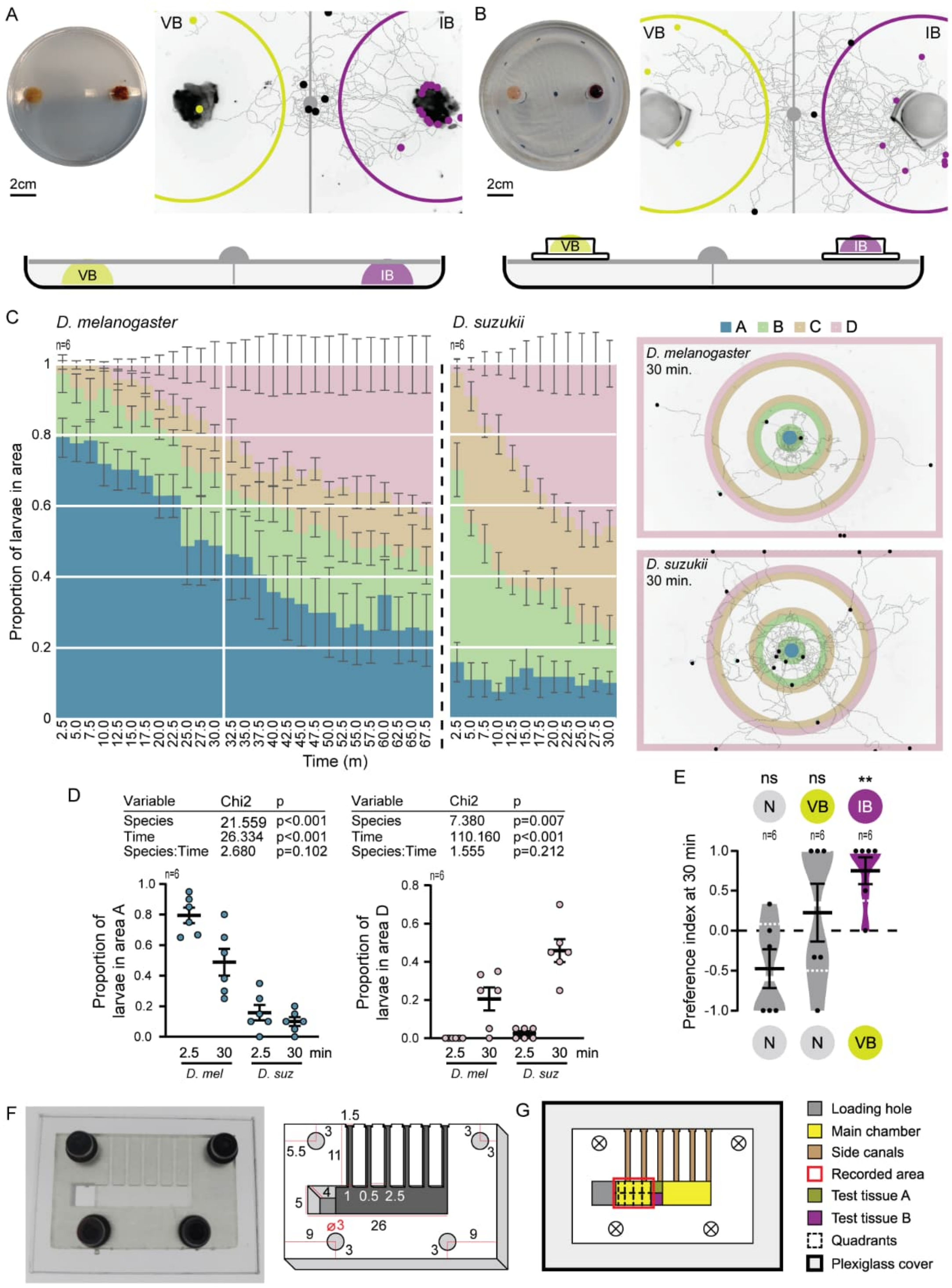

*Figure S3. Choice assays, related to Figure 3. A. Top left: Picture of the agarose gel with embedded fruit tissue samples. Top right: Example of automatically tracked D. suzukii larvae on a gel with embedded fruit tissues. Bottom: Schematic side view of the placement of tissue samples (green and purple semicircles) embedded in the agarose gel. B. Top left: Picture of the agarose gel 1.5 ml Eppendorf tube caps with fruit tissue samples. Top right: Example of automatically tracked D. suzukii larvae on a gel with fruit tissues in 1.5 ml Eppendorf tube caps. Bottom: Schematic side view of the placement of tissue samples (green and purple semicircles) in Eppendorf tube caps on top of the agarose gel surface. In both panels, left side: fresh fruit tissue; right side: D. suzukii larva modified tissue. C. Left: Mean proportion (± SEM) of D. melanogaster (left) and D. suzukii (right) larvae present in each area of empty arenas at each time point in 2.5 min intervals. n: number of observed arenas with 20 larvae. Right: Example pictures of automatically tracked D. melanogaster (top) and D. suzukii (bottom) at the 30 min time point showing the quantified areas. D. Proportion of D. melanogaster and D. suzukii larvae in area A (left) and in areas D (right) at the 2.5 and 30 min time points in empty arenas were analyzed with binomial generalized linear mixed models (binomial glmm). n: number of observed arenas with 20 larvae. Given are means ± SEM. Dots represent individual data points. E. Preference index at 30 min of first instar D. melanogaster larvae in choice assays towards agarose-embedded fruit tissue. N=nothing, VB=virgin blueberry, IB=D. suzukii larval-modified blueberry. Student t-Test against a hypothetical media = 0, N v N, $t = 1.961$. N v VB, $t = 0.6143$. N v IB, $t = 4.392$. White line in the violin plot: Median, Black dots: Individual values, Black line: Mean, Error bars: SEM, n = number of assays, ns: non-statistically significant differences, **: statistically significant at $p<0.01$. F. Left: Picture of the assembled apparatus for digging choice assays. Middle: Schematics of the 3D printed part of the apparatus, sizes expressed in mm. Right: Schematics of the digging choice assay apparatus setup for experiment and division of the space for quantification. Larvae were assayed in the main chamber, starting on the right edge of the loading hole and digging towards the right. The recorded area (red square) and the quantification quadrants (dashed lines) are pictured in Figure 3K. When preparing the apparatus for the assays, tissues are placed in the recessed main chamber (yellow rectangle) of the 3D printed chip (positions marked by green and purple squares). Then it is covered with a Plexiglas transparent piece (gray rectangle with thick black line) tightened with bolts, nuts and washers (crossed circles). Agarose is pipetted hot through the loading hole (dark gray square), filling the main chamber. The side canals (orange) allow air to be displaced from the main chamber as agarose fills it.*
